# Developing capacity for implementation and evaluation of vaccine trials in Uganda: Perspective of the Makerere University Walter Reed Project

**DOI:** 10.4314/ahs.v22i2.6S

**Published:** 2022-08

**Authors:** Prossy Naluyima, Betty Mwesigwa, Allan Tindikahwa, Stephen Mugamba, Jude Thaddeus Ssensamba, Ezra Musingye, Grace Mirembe, Hannah Kibuuka, Fred Wabwire-Mangen

**Affiliations:** 1 Makerere University Walter Reed Project; 2 School of Public Health, College of Health Sciences, Makerere University

**Keywords:** Vaccines, community participation, developing countries

## Abstract

**Introduction:**

Infectious diseases and neglected tropical diseases continue to be a major challenge in resource limited settings, causing significant morbidity and mortality. Although vaccines are a key biomedical prevention tool, resource limited settings often lack the infrastructure, regulatory frameworks, and skilled human resource to conduct vaccine clinical trials. To address this gap, the Makerere University Walter Reed Project (MUWRP) was established and has contributed to vaccine research in Uganda and globally.

**Methods:**

This was achieved through training a strong vaccine clinical trial workforce; development of requisite clinical trial infrastructure for research activities and management of investigational products; conducting phase I-III vaccine trials and contribution to national ethical and regulatory frameworks that protect participants.

**Results:**

As of 2022, MUWRP had successfully conducted and completed five phase I/II HIV vaccine clinical trials, five for Ebola and Marburg, while one phase I/II Schistosomiasis and one phase III COVTD-19 vaccine clinical trial are ongoing.

**Discussion:**

The completed vaccine trials provided critical scientific knowledge on the safety and immunogenicity of investigational products which informed the design of better vaccines for diseases of global health importance.

**Conclusion:**

Academia, through establishment of appropriate partnerships can contribute to the identification of solutions to complex public health challenges.

## Introduction

The development of safe and effective vaccines has been one of the most significant advances in the modern history of medicine. As of December 2021, there were vaccines to 25 infectious diseases^1^. However, there is an urgent need to develop vaccines for prevalent, endemic diseases like HIV, malaria and schistosomiasis, and emerging and re-emerging infections in Africa. The COVID-19 pandemic further underscored the relevance of vaccine development for the prevention of infectious diseases of public health concern^2^. That said, there is a paucity of information on developing capability for vaccine research in sub-Saharan Africa^3^.

In the late 1980s, Uganda had one of the highest prevalence of HIV in the world^4^–^6^. Although numerous public health strategies led to almost 10% reduction in HIV prevalence by the late 1990s, it remained unacceptably high making HIV vaccine research a priority for Uganda^4^,^7^,^8^. In February 1996 the Minister for Health, while addressing an international network of scientists^9^, stated Uganda's readiness to participate in HIV vaccine clinical trials. The ALVAC (clade B) vCP205 was the first HIV vaccine clinical trial in Uganda and Africa, and formed the foundation for vaccine research in Uganda^4^, ^10^.

In 1998 Makerere University and the Rakai Health Sciences Program partnered with the US Military HIV Research Program (MHRP) and the Henry M. Jackson Foundation for the Advancement of Military Medicine to study the molecular and epidemiological evolution of HIV in preparation for potential vaccine clinical trials (VCTs). This effort led to the establishment of the Makerere University Walter Reed Project (MUWRP) in 2002, to build local capacity to conduct HIV vaccine trials. MU-WRP later evolved to other VCTs for Ebola, Marburg, Schistosomiasis, and COVID-19. This article describes the development of MUWRP's capacity and capability to conduct VCTs, and its contribution to vaccine research in Uganda through Makerere University College of Health Sciences (MakCHS).

## Methods

### About MUWRP

MUWRP is a non-profit biomedical research organization, whose mission is to mitigate disease threats through quality research, health care and disease surveillance. The project's scope includes clinical research; the Presidential Emergency Program for AIDS Relief funded comprehensive HIV prevention, care and treatment program; the Emerging Infectious Diseases Program whose role is surveillance, one health and global health security; and the Joint Mobile Emerging Disease Intervention Clinical Capability program whose primary objective is to establish capacity for clinical research during filovirus outbreaks. To achieve its current VCTs capability, MUWRP created a strong clinical trial workforce; developed critical infrastructure for research activities, data management, information technology, laboratory testing and storage; aligned with international financial standards; created community research awareness, buy in and political will; and instituted ethical and regulatory frameworks that protect participants. Hereunder we describe how these capabilities were achieved.

### Human Resource Development

MUWRP conducts VCTs to internationally recognized standards in order to generate credible data that informs prioritization of biological products^11^. To achieve this, MUWRP developed a diverse workforce including physicians, public health specialists, medical officers, research nurses, laboratory scientists, clinical research coordinators, clinical trial administrators, records officers, research pharmacists, quality and compliance specialists, data management staff, and grants managers for clinical trial implementation. Training included general clinical trial training, Good Clinical Practice (GCP), Good Clinical Laboratory Practice (GCLP), Human Subjects' Protection (HSP), Good Documentation Practice and biosafety, all to the standard of International Council for Harmonization of Technical Requirements for Pharmaceuticals for Human Use (ICH)^12^. Regular competence assessments to ensure maintenance of these skills are conducted throughout the period of employment. Collectively the staff have research experience ranging from two to over 19 years.

### Establishing VCT Infrastructure

MUWRP's infrastructure development was guided by ICH and international partners. Initial space allocation at the MakCHS Pathology building was insufficient to accommodate the research clinic, laboratory and administrative offices. MUWRP relocated the research clinic and administrative offices to Plot 42, Nakasero Road. The research clinic has 12 well-equipped clinic rooms for private physical examination and counselling, a well-equipped phlebotomy room, a waiting area, plus secure records and data offices with restricted biometric access^13^. A research pharmacy equipped with a biosafety level II (BSL-2) cabinet, 4 freezers (-80°C and -40°C) and 2 refrigerators monitored by digital thermometers with temperature and humidity data loggers was established. It manages investigational products according to national and international regulations including National Drug Authority (NDA) and Food and Drug Authority (FDA, U.S.). Access to the pharmacy is restricted by key and biometrics.

### Data Management and IT Infrastructure

Integrity, safety, and validity of trial data is critical to the evaluation of vaccines. MUWRP instituted a data management center (DMC) equipped to securely access, store and process vaccine trial data according to Health Insurance Portability and Accountability Act (HIPAA) privacy guidelines. The Heating, Ventilation and Air Conditioning Controlled DMC has a Tier II Access with Virtualized Enterprise grade core servers and clusters. A secure Storage Area Network that hosts application, file shares, relational databases and synchronously replicates data backups across redundant secondary sites was also developed. Investments in a defense in-depth approach which secures all mobile devices, workstations and servers through endpoint signature-based security, web proxies and firewalls were made. MUWRP also instituted high standards in VCTs data capture, processing and quality control by training data entry and management specialists; acquiring analytical infrastructure and software (e.g., OpenClinica [OpenClinica LLC] and STATA [StataCorp, College Station, TX, USA], respectively); and rolling out the Clinic Appointment Scheduling & Tracking (CAST) system which tracks attendance, scheduling and follow-up for vaccine trial participants.

### Laboratory Capacity

Laboratory capacity for moderate and highly complex testing is a challenge in resource limited settings^14^–^16^. MUWRP established its clinical and research BSL-2 laboratory at the MakCHS and immediately instituted processes leading to College of American Pathologists' accreditation in 2005. This accreditation has been maintained through a rigorous continuous quality assurance and quality improvement program, based on GCLP^17^. The laboratory has capacity for safety tests, light microscopy, diagnostic immunology, cellular immunology, molecular diagnostics, and biological specimen processing and cryopreservation. A robust laboratory information management system, plus a biorepository with over thirty -80°C and liquid nitrogen freezers supported by 2 liquid nitrogen plants (400 liters/day) and two back-up generators were established. There is in-house capacity for shipping specimens for complex testing following International Air Transport Association standards. All laboratory work is handled by a diverse team of well-trained and certified laboratory scientists and technologists.

### Financial Management Capability

Another important aspect of vaccine research is the financial capability of institutions to manage grants^18^ and procure supplies. MUWRP invested in a robust accounting system that supports budgeting, expenditure tracking, forecasting and reporting per funder requirements. MUWRP further adheres to Generally Accepted Accounting Principles and cost principles of (U.S.) federal contracting that meet the standards of the Comptroller General of the US Government, and is regularly audited by internal and external auditors. Relatedly, MUWRP developed a supply chain management system that adheres to internationally acceptable procurement principles and practices in accordance with the Federal Acquisitions Regulations of the U.S.

### Community engagement

Identification of safe and effective vaccines requires full participation of communities most affected by the disease in question^19^. Community engagement entails research education, efforts to allay fears and correct myths and misconceptions, and winning community trust to create partnerships in vaccine development^20^–^22^. MUWRP's community engagement office utilizes platforms where members of the community, policy makers and researchers work together to address health-related issues. MUWRP further formed a community advisory board with representation from former trial participants, religious leaders, the media, politicians, civil society, and key and vulnerable populations, who meet regularly to review the trial protocols and ensure that the scientific priorities are relevant to the population and community voices are heard. Policy makers and politicians are engaged on the importance of vaccine research in the country (Picture 1). When a clinical trial requires participation of vulnerable populations, peer leaders support recruitment and retention. Table 1 highlights some of the community engagement challenges and their solutions.

**Table 1:** Challenges of community engagement

Challenge	Solution
Myths and misconceptions about vaccine clinical trials in general, especially vaccine safety	Community education, engagement of opinion leaders, and advocacy
Numerous trial follow-up visits	Participant education on the importance of follow-up visits and collection of contact and locator information
Access to key populations such as commercial sex workers	Use of sub-advisory boards that are population- focused; an improvement from the snowballing mechanisms of identifying potential participants
Participant expectations for healthcare beyond the provisions and lifetime of the trial	Continuous participant education on the trial requirements, provisions and limitations

### Instituting an Ethical and Regulatory Framework for VCTs

The first HIV vaccine clinical trial conducted in Uganda and Africa raised many questions concerning the availability of an ethical and regulatory framework for equity in biomedical research in Uganda^4^, ^10^, ^23^, ^24^. MUWRP set up a regulatory, quality and compliance department to ensure trial compliance with local (Research Ethics Committees, Uganda National Council for Science and Technology [UNCST], NDA) and international (ICH, GCP, etc.) guidelines for research involving human subjects. This department scrutinizes protocols prior to submission for review to national regulatory authorities, tracks and follows up all regulatory correspondence, internally monitors trials, reports adverse events and deviations, organizes continual staff trainings, submits annual progress reports, obtains subsequent trial approvals in a timely manner, and maintains all pertinent documentation. All these processes are guided by a quality management plan and internally developed SOPs.

### Training on laboratory research and quality systems

MUWRP offers internships to students and graduates from Makerere and other universities on laboratory processes, GCLP, biosafety, among others. Students are actively supported to utilize the laboratory infrastructure for postgraduate research, including basic sciences projects. In addition, the laboratory conducts didactic and hands-on training on GCLP for other clinical research sites (CRS) and private health facilities in Uganda.

## Results

In light of the above processes, MUWRP has grown into a model VCT site. To date, MUWRP has supported 12 master of science and 3 doctor of philosophy students, and has supported the improvement of quality management systems in two regional referral hospitals (Fort Portal and Kayunga), leading to laboratory accreditation^25^. In addition, MUWRP has successfully conducted 12 Phase I/II vaccine clinical trials for HIV, Ebola, Marburg, and Schistosomiasis, and a Phase III COVID-19 vaccine trial. Hereunder is a summary of some of the VCTs MUWRP has conducted and their key outcomes.

*RV 156, 2004*

This was MUWRP's first phase I vaccine trial which evaluated the safety and immunogenicity of a multiclade HIV-1 DNA plasmid vaccine, VRC-HIVDNA009-00 VP The trial (NCT01549470) enrolled healthy HIV negative adults 18–50 years in Kampala, Uganda. The product was well-tolerated and this trial laid the foundation for design and conduct of subsequent HIV vaccine clinical trials.

*RV 172, 2006*

This phase I/II clinical trial (NCT00123968) evaluated the safety and immunogenicity of a multiclade HIV-1 DNA plasmid vaccine, VRCHIVDNA016-00-VP, boosted by a multiclade HIV-1 recombinant adenovirus-5 vector vaccine, VRC-HIVADV014-00-VP, in HIV uninfected adults in East Africa. The vaccine was safe and well tolerated. HIV-specific T cell responses were detected in 63% of vaccinees, with titers of preexisting Adenovirus serotype 5 (Ad5) neutralizing antibody not affecting the frequency and magnitude of T cell responses in prime-boost recipients^26^. The vaccines were further tested in unique subpopulations in a phase 2b trial in the US to safely evaluate their efficacy^27^.

*RV 247, 2009*

This Phase lb trial (NCT00997607) evaluated the safety and immunogenicity of an Ebola DNA Plasmid Vaccine, VRC-EBODNA023-00-VP, and a Marburg DNA Plasmid Vaccine, VRC-MARDNA025-00-VP in healthy HIV negative adults aged 18–50 years. This trial was the first Ebola or Marburg vaccine clinical trial in Africa, and the results showed that, given separately or together, both vaccines were well tolerated and elicited antigen-specific humoral and cellular immune responses, thus contributing to expedited development of more potent Ebola virus vaccines that use the same wild-type glycoprotein antigens^28^.

*RV 262, 2012*

The phase I HIV vaccine trial evaluated the safety and immunogenicity of PENNVAK™-G DNA (ENV & GAG) administered by intramuscular Biojector® 2000 or Cellectra® intramuscular electroporation device followed by MVA-CMDR (HIV-1 cm235 ENV/cm240 gag/pol) boost in healthy, HIV uninfected adults in Kampala. This trial (NCT01082692) also tested if the mode of vaccine administration influences immune responses. Results showed that cellular responses were observed in 57% of vaccine recipients tested and were CD4 predominant, including high rates of binding antibody responses to CRF01_AE antigens. Electroporation did not confer advantage over needle-free device delivery of the DNA prime for this regimen^29^.

*RV417/HIV-V-A004, 2015*

This was a Phase I/IIa trial (NCT02315703) to evaluate the safety/tolerability and immunogenicity of homologous Ad26 Mosaic Vector Vaccine Regimens or Ad26 Mosaic and Modified Vaccinia Ankara Mosaic Heterologous Vector Vaccine Regimens, with high dose, low-dose or no Clade C gpl40 protein plus adjuvant for HIV prevention in healthy Ugandans. This was a complex trial where eligible participants were randomized into one of eight trial arms at 12 clinical sites. The vaccine regimens demonstrated favorable safety and tolerability. The mosaic Ad26 prime/Ad26+gpl40 boost regimen was the most immunogenic in humans; it elicited env-specific bindin-antibody responses, antibody-dependent cellular phago cytosis responses, and T-cell responses in 100%, 80% and 83% of vaccine recipients, respectively, and was evaluated further in follow-on HIV vaccine clinical trials^30^.

*EBOVAC2/VAC52150EBL2002, 2015*

This phase Ib, clinical trial (NCT02564523) evaluated the safety, tolerability and immunogenicity of the Ebola Ad26.ZEBOV and MVA-BN-Filo in healthy adults (cohort 1), HIV infected adults, adolescents (cohort 2) and children (cohort 3) in multiple African sites including MUWRP. Results showed that Ad26.ZEBOV, MVA-BN-filo vaccination was well tolerated and immunogenic in HIV uninfected and HIV-infected African adults, plus adolescents and children. Increasing the interval between vaccinations from 28 to 56 days improved the magnitude of humoral immune responses. Antibody levels persisted to at least 1 year, and Ad26.ZEBOV booster vaccination demonstrated the presence of vaccination-induced immune memory. The 56 day regimen was approved by the European Union for prophylaxis against Ebola (Zaire) disease in adults and children ≥1 year of age^31^.

*RV 508, 2019*

This phase 1 trial (NCT02231866) tested the safety, tolerability and immunogenicity of two doses of an Ebola Sudan Chimpanzee adenovirus vector vaccine, VRC-EBOADC086-00-VP (cAd3-EBOVS), in healthy adults 18–50 years in Kampala. This clinical trial was the first to evaluate a stand-alone Ebola Sudan strain vaccine. Sample and data analysis are underway.

*TSP-18-03, 2019*

This Phase I/II trial (NCT03910972) is evaluating the safely, immunogenicity and efficacy of Sm-TSP-2/Alh-drogel® with or without AP10-701 for intestinal Schis tosomiasis in healthy Ugandan adults aged 18 to 45 years in Kampala. It is the first schistosomiasis vaccine clinical trial in Africa. Phase I enrolled 90 participants, while the ongoing phase II of the trial seeks to enroll 200 participants infected with S. mansoni at screening and seeks to evaluate vaccine efficacy post treatment with praziquantel. This trial is critical to the development of an effective vaccine for Schistosomiasis, a neglected tropical disease that continues to afflict the poor in the developing world.

*VAT00008, 2021*

This is a parallel-group, Phase III, multi-stage, modified double-blind, multi-armed trial (NCT04904549) to assess the efficacy, safely, and immunogenicity of two SARS-CoV-2 Adjuvanted Recombinant Protein Vaccines (monovalent and bivalent) for prevention against COVID-19 in adults 18 years of age and older. The trial targeted COVID-19 naive and exposed, healthy and COVID-19 high risk persons. Enrollment commenced in December 2021 and participant follow-up is ongoing.

## Discussion

Vaccines remain the cornerstone for prevention of infectious diseases. The current efforts to reach herd immunity for COVID-19 underline the importance of vaccine research in addressing national and global public health threats. There is need for stronger vaccine trial capability in resource limited settings for effective response to emerging and re-emerging infectious disease threats. This article has highlighted MUWRP's progress towards setting up VCT capability in a resource limited setting and discusses below, the impact of these initiatives towards national and global efforts in prevention of infectious diseases.

### Impact in Uganda and globally

Although the majority of vaccine clinical trials presented are early phase trials, the knowledge attained has contributed tremendously to the field of vaccine development. The safely and immunogenicity data from the first Ebola/Marburg vaccine trial provided a basis for the viral vectored vaccines that were tested during the 2014–2016 Ebola virus disease outbreak in West Africa^32^. The EB-OVAC2/VAC52150EBL2002 trial vaccines subsequently tested at two CRS in Uganda (including MUWRP)^31^ were approved by the European Union for prophylactic use during Ebola outbreaks. In addition, safety and immunogenicity data from HIV vaccine clinical trials at MUWRP have contributed to the body of knowledge guiding the design of investigational products for the prevention^26^ and treatment^33^ of HIV infection. MUWRP is currently testing the first vaccine in Africa against schistosomiasis and a phase III COVID-19 vaccine that may address the critical need for new preventive interventions.

### Contribution to Building Capacity of other Vaccine Research Institutions in Uganda

MUWRP has contributed to the critical mass of clinical, laboratory and sociological researchers in the country. Many staff who trained and worked at MUWRP have moved on to successfully set-up or support other CRSs, establishing a foundation for locally designed research protocols. Staff actively contributed to the development of the National Guidelines for Research involving Humans as Research Participants, the Uganda National Health Laboratory Services policy and the national bio-safety and biosecurity guidelines and regulations. MUWRP has contributed to the body of scientific knowledge through presentations at national and international scientific meetings, with a total of six publications^26^, ^28^, ^30^, ^31^, ^34^, ^35^ with two as first author publications^26^, ^28^ generated from vaccine clinical trials. In addition, staff are members of key Ministry of Health technical working groups that develop guidelines for improved health care. Other engagements within and outside Makerere University have resulted in collaborations with both local and international colleges, universities, pharmaceutical and research bodies that have created new training and research opportunities for scientists in Uganda. MUWRP's state-of-the-art infrastructure continues to build national capacity and capability for the conduct of complex vaccine trials through training, mentorship and internships.

## Conclusion

Infectious diseases continue to pose a significant threat to global health and productivity. VCTs help in the identification of vaccine products for the prevention of diseases, but the lack of adequate infrastructure, skilled personnel and effective ethical and regulatory frameworks may limit their conduct in low resource settings. This article summarizes the efforts and results of building local capacity and capabilities for the successful conduct of impactful VCTs in a resource limited setting. MUWRP demonstrates how partnerships between academia (MAKCHS) and non-academic organizations can develop skills, infrastructure and frameworks for the evaluation of appropriate solutions to infectious disease threats.

## Figures and Tables

**Picture 1:**
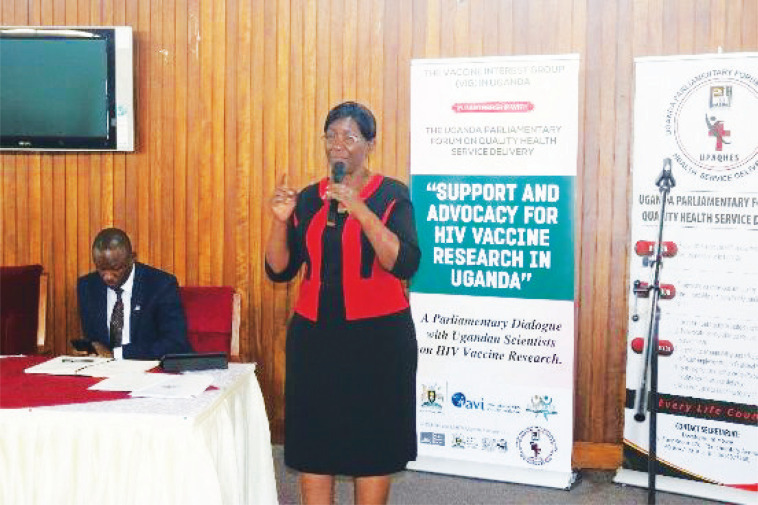
Dr. Kibuuka (Executive Director) addressing parliamentarians about HIV vaccine research
